# Initiatives promoting planetary health education in Germany: An overview

**DOI:** 10.3205/zma001620

**Published:** 2023-05-15

**Authors:** Eva-Maria Schwienhorst-Stich, Katharina Wabnitz, Eva Geck, Sophie Gepp, Laura Jung, Anna Mumm, Jörg Schmid, Anne Simmenroth, Johanna Simon, Michael Eichinger

**Affiliations:** 1 University Hospital Würzburg, Department of General Practice, Würzburg, Germany; 2 University of Würzburg, Teaching Clinic of the Faculty of Medicine & University Hospital Würzburg, Institute of Medical Teaching and Medical Education Research, Würzburg, Germany; 3 Ludwig-Maximilians-University Munich, Chair of Public Health and Health Services Research, Institute for Medical Information Processing, Biometry and Epidemiology (IBE), Munich, Germany; 4 Centre for Planetary Health Policy (CPHP), Berlin, Germany; 5 University of Würzburg, Würzburg, Germany; 6 Charité – Universitätsmedizin Berlin, corporate member of Freie Universität Berlin and Humboldt-Universität zu Berlin, Berlin, Germany; 7 German Alliance on Climate Change and Health (KLUG), Berlin, Germany; 8 Leipzig University Medical Center, Division of Infectious Diseases and Tropical Medicine, Leipzig, Germany; 9 University of Ulm, Ulm, Germany; 10 Heidelberg University, Medical Faculty Mannheim, Center for Preventive Medicine and Digital Health, Mannheim, Germany; 11 University Medical Center of the Johannes Gutenberg-University Mainz, Institute of Medical Biostatistics, Epidemiology and Informatics, Mainz, Germany

**Keywords:** planetary health, planetary health education, climate change, sustainable healthcare, transformative education

## Abstract

Planetary health education focuses on the climate and ecological crises and their adverse health effects. Given the acceleration of these crises, nationwide integration of planetary health education into undergraduate and graduate education, postgraduate training and continuing education for all health professionals has repeatedly been called for. Since 2019, planetary health education has been promoted by several national initiatives in Germany that are summarized in this commentary:

1. National Working Group Planetary Health Education,

2. Manual for planetary health education,

3. Catalog of National Planetary Health Learning Objectives in the National Competency-Based Catalog of Learning Objectives for Medical Education,

4. Working Group Climate, Environment and Health Impact Assessment at the Institute for Medical and Pharmaceutical Examinations,

5. Planetary Health Report Card, and

6. PlanetMedEd study: planetary health education in medical schools in Germany.

We hope these initiatives promote collaboration across institutions involved in educating and training health professionals, inter-professional cooperation as well as rapid implementation of planetary health education.

## Abstract (de)

Lehre zu Planetary Health befasst sich mit den Zusammenhängen zwischen der Klima- und Umweltkrise und der menschlichen Gesundheit. Mit Fortschreiten der Krise mehren sich die Rufe nach flächendeckender Integration von Lehre zu Planetary Health in die Aus-, Weiter- und Fortbildung aller Gesundheitsberufe. Die flächendeckende Integration wird seit 2019 durch mehrere bundesweite Initiativen unterstützt, die im folgenden Kommentar exemplarisch vorgestellt werden:

1. AG Planetary Health Lehre,

2. Leitfaden für Lehrangebote zu Planetary Health,

3. Themen- und Fachkatalog Planetare und Globale Gesundheit im Nationalen Kompetenzbasierten Lernzielkatalog Medizin,

4. AG Klima, Umwelt und gesundheitliche Folgenabschätzung am Institut für Medizinische und Pharmazeutische Prüfungsfragen,

5. Planetary Health Report Card und

6. PlanetMedEd Studie: Lehre zu Planetary Health an Medizinischen Fakultäten in Deutschland.

Die bundesweiten Initiativen sollen einen Beitrag zu standortübergreifender Zusammenarbeit, interprofessioneller Ausrichtung und rascher Implementierung von Lehre zu Planetary Health leisten.

## Introduction

Planetary health education focuses on the climate and ecological crises and their adverse health effects [1]. Given the potential of health professionals to contribute to climate change mitigation, environmental protection and climate adaptation, nationwide integration of planetary health education into undergraduate and graduate education, postgraduate training and continuing education for all health professionals has been repeatedly called for [2], [3], [4], [5], [6], [7], first studies show the demand of the students [8]. Several initiatives have been launched in Germany since 2019 to support these efforts. In this commentary, we provide an overview of selected national initiatives including contact information and recommendations for further reading.

## Six selected national initiatives

### 1. National Working Group Planetary Health Education

The inter-professional working group, jointly hosted by the German Alliance on Climate Change and Health and Health for Future, was founded in February 2021 to provide a platform for knowledge exchange targeting all health professionals involved in planetary health education. The working group focuses on different aspects of planetary health education including presentations of innovative modular teaching materials and syllabi of elective courses as well as journal clubs covering seminal contributions to the field of planetary health education. The WeChange platform is used to network and to collect material pertinent to planetary health education.

Sign up to the mailing list: [https://www.klimawandel-gesundheit.de/ag-lehre-mailingliste/]

Sign up to the WeChange platform: [https://wechange.de/project/ag-planetary-health-lehre/]

#### 2. Manual for planetary health education

In 2021, members of the national working group developed a manual for planetary health education targeting both staff and students at universities and universities of applied sciences involved in teaching. On 50 pages, the authors present, *inter alia*, background information on planetary health education, didactic principles, best-practice examples and recommendations for further reading. Given its hands-on approach, the manual supports the integration of planetary health education in institutions of higher education. For example, the manual provides suggestions on how to integrate content of the Planetary Health Academy [9] of the German Alliance on Climate Change and Health, including slides and video recordings of lectures, into planetary health education [https://planetary-health-academy.de/]. The latest version of the manual is available online [10].

#### 3. Catalog of National Planetary Health Learning Objectives in the National Competency-Based Catalog of Learning Objectives for Medical Education 

The Catalog of National Planetary Health Learning Objectives [11] was developed between 2019-2021 as part of a nationwide multi-stakeholder process to refine the Competency-based Catalog of Learning Objectives [https://www.nklm.de]. The catalog comprises learning objectives on clinical competencies for climate adaptation and climate-sensitive anticipatory guidance as well as skills supporting physicians in initiating and implementing change processes outside patient care that contribute to climate mitigation and sustainability. To promote the integration of planetary health education in all areas of medical education, the catalog contains cross references to the main chapters of the Competency-Based Catalog of Learning Objectives for Medical Education. The planetary health learning objectives can guide, *inter alia*, the development of longitudinal planetary health curricula or focus areas within medical curricula stipulated in the current version of the updated medical licensing regulations for Germany.

#### 4. Working Group Climate, Environment and Health Impact Assessment at the Institute for Medical and Pharmaceutical Examinations 

The Institute for Medical and Pharmaceutical Examinations, developing and administering centralized state examinations for medicine, dentistry, pharmacology, and psychotherapy in Germany, founded the working group in January 2021. The inter-professional working group comprises external experts and aims at adding aspects of planetary health education to the syllabi of the state examinations.

#### 5. Planetary Health Report Card

In this international initiative, medical students evaluate their medical schools based on standardized planetary health indicators covering the following areas:

1. planetary health curriculum,
2. interdisciplinary research,
3. institutional support for student-led initiatives,
4. community outreach and advocacy, and
5. campus sustainability.

While previously restricted to the USA, Canada, UK and Ireland, several medical schools in Germany have been evaluated based on the report card since 2021. The extension of the report card to curricula of other health professionals is currently prepared by the international PHRC team. The results of the assessment are published online on Earth Day every year: [https://phreportcard.org/]. 

Contact details for Germany: joergschmid@posteo.de

#### 6. PlanetMedEd study: planetary health education in medical schools in Germany

Since 2021, researchers at Würzburg University and Heidelberg University conduct the PlanetMedEd study. The study comprises three parts and aims to provide an overview of planetary health education currently provided by medical schools in Germany (part A), to investigate demand for planetary health education among medical students (part B) and to explore barriers and enabling factors for the implementation of planetary health education in medical schools (part C).

[https://www.med.uni-wuerzburg.de/planetaregesundheit/aktivitaeten/forschungsprojekte/] 

Contact information for part A and C: schwienhor_e@ukw.de, for part B: michael.eichinger@medma.uni-heidelberg.de

## Conclusions

To fully harness the transformative potential of health professionals for climate change mitigation, environmental protection and climate adaptation, it is crucial to integrate planetary health education into undergraduate and graduate education, postgraduate training and continuing education for all health professionals. We hope that the national initiatives summarized in this commentary will contribute to the implementation of planetary health education in medical schools and to support cooperation across institutions by describing the status quo, highlighting unmet needs, providing networking opportunities and promoting the development of teaching resources.

Complementing the national initiatives presented here, planetary health education could be promoted by several other measures. National initiatives currently focusing on medical education could be adapted and extended to other health professions. Networking among initiatives could contribute to inter-professional collaboration that is instrumental in addressing the substantial adverse health effects of the climate and ecological crises. Comparable to the Competency-Based Catalog of Learning Objectives for Medical Education, planetary health education should be integrated in other legal documents including the updated medical licensing regulations and learning objectives of other health professions. Complementing the development of teaching materials for students, lecturers should be supported in expanding their expertise in planetary health and their didactic skills to deliver high-quality transformative teaching [2], [3], [12].

## Competing interests

The authors declare that they have no competing interests. 
